# Thyroid Function in Male Infertility

**DOI:** 10.3389/fendo.2013.00174

**Published:** 2013-11-13

**Authors:** Elzbieta Krajewska-Kulak, Pallav Sengupta

**Affiliations:** ^1^Integrated Medical Care, Medical University of Bialystok, Bialystok, Poland; ^2^Department of Physiology, Vidyasagar College for Women, University of Calcutta, Calcutta, West Bengal, India

**Keywords:** infertility, male reproduction, spermatogenesis, testosterone, thyroid hormones, TSH

Thyroid gland, previously supposed not to have any impact on spermatogenesis and male fertility, are now being recognized as having important role in male reproductive functions. Most of the studies on the effect of thyroid hormones on male fertility were conducted between the years 1970 and 2000 (1). The effects of thyroid hormone alterations on the reproductive system have been studied extensively in human subjects and animal models that have generally shown that changes from normal thyroid function resulted in decreased sexual activity and fertility (2, 3). The underlying mechanisms, however, are not constant throughout all species, and results from different studies disagree (4).

In rats rendered thyrotoxic by T4 resulted in decreased serum gonadotropin levels (5), decrease in total lipids, cholesterol, and phospholipids in testes, and synthesize increased amounts of testosterone (6). In immature male mice aged less than 4 weeks, the administration of slightly supra-physiological T4 doses resulted in a tendency toward early maturation and shortening of development period. Conversely, larger TH doses resulted in decreased testes weights and seminal vesicles, both in mice and rabbits (3). Direct effects of T4 resulted in minimal oxygen consumption changes in testes when T4 was present in testicular slice incubations (7). Finally, the effects of T4 on spermatogenesis are conflicting (8), but it would appear that T4 does not exert a direct effect on spermatogenesis in mature rats or rams (9). In rats, T3 affects testis maturation, and thyroid receptor (TR) type-1 (TR-1) expression in rats’ testes (10, 11). Maximal Sertoli cell proliferation coincides with maximal T3 binding capacity in testis, suggesting that the main target of T3 action is the Sertoli cell. However, T3 also plays a significant role in differentiation of the seminiferous epithelium, and studies in rodents have shown that T3 is an important factor in maturation of Leydig cells. The presence of T3 is necessary to initiate differentiation of mesenchymal cells into Leydig progenitor cells, and T3 works in concert with other hormones [luteinizing hormone (LH) and IGF-I] to promote Leydig cell development (12). Data from other animal species (such as deer, sheep, cattle, birds, and mink) also suggest that T3 is a component of the neuroendocrine system that regulates seasonal cycles of reproductive activity (13). The underlying mechanisms postulate that T3 triggers cessation of reproduction at the end of the reproduction season because circulating T3 levels in deer rise at the time of seasonal transition to the non-breeding state and thyroidectomy results in the absence of seasonal regression of the testis (14, 15). Hypothyroidism induced or occurring soon after birth was associated with marked sexual maturation and development delays in animals (16). Rats made hypothyroid transiently by propylthiouracil (PTU) administration showed a decrease in testicular size, retardation in Sertoli cell differentiation, and prolongation of Sertoli cell proliferation time (17). When the rats became older and returned to a euthyroid status, there was an increase in testis size, Sertoli cell number, and sperm production (18). In other studies where experimental hypothyroidism in rats and rams was left untreated for more than 1 month, there was an arrest of sexual maturity, decreased testosterone concentration as well as an absence of libido and ejaculate (6, 19). It would therefore appear that hypothyroidism affects the immature, but not the mature, testis. Pekary and Sattin (20) showed that both hypothyroidism and castration reduced TRH levels (20).

The two most common types of thyroid diseases are hypothyroidism and hyperthyroidism. Studies assessing the role of hypo- and hyperthyroidism in male infertility have also been conducted in human subjects. Hypothyroidism may result in a decrease in the sex hormone binding globulin (SHBG) levels and a decrease in total serum testosterone levels, as well as a decrease in the LH and the follicle stimulating hormone (FSH) levels (21). In cases of prolonged pre-pubertal hypothyroidism due to drop in LH and FSH levels, the Leydig and Sertoli cells, respectively are less stimulated to differentiate into mature cells, negatively affecting spermatogenesis. This increases the number of cells in the testes but decreases the number of mature cells. Thus, in patients with hypothyroidism, increased testicular size is observed along with a significant drop in mature germ cells within the seminiferous tubules (22, 23). Fortunately, hypothyroidism is very rare in males with an occurrence rate of only 0.1% in the general population (21). Among the studies on human subjects, Corrales Hernandez et al. (24) analyzed blood and semen samples of patients with primary hypothyroidism (24). The study concluded that hypothyroidism adversely affected semen quality by compromising semen volume and progressive sperm motility. Krassas et al. (25) conducted another study on human subjects with hypothyroidism (25). The authors reported abnormal sperm morphology and decreased motility in the patients. It is therefore evident that hypothyroidism adversely affects male fertility. Similarly, all the studies on hyperthyroidism also reported adverse effects on male reproductive organs and fertility. Clyde et al. by studying individual cases reported adverse effects of hyperthyroidism on semen quality (26). Clyde looked at three individual case studies of men with hyperthyroidism and infertility. Hormone levels were measured and recorded, and the overall results indicated that all three patients had low sperm counts as well as decreased sperm motility. However, such abnormalities were corrected when the patients were treated for thyroid disease. Therefore, Clyde concluded that male infertility is more common than previously thought in males with hyperthyroidism, possibly in correlation with elevated levels of testosterone, LH, and FSH. Hudson and Edwards (27) after conducting study on human subjects stated adverse effects of hyperthyroidism on spermatogenesis by altering sex steroid levels (27). Similarly, Krassas and Perros claimed adverse effects of hyperthyroidism on seminal parameters of human subjects (21). Most of the studies concerning hyperthyroidism were conducted on human subjects with only one conducted on rats. Rijntjes et al., in their study on rats concluded that hyperthyroidism delays Leydig cell development and adversely affects spermatogenesis (28).
